# Assessing brain immune activation in psychiatric disorders: clinical and preclinical PET imaging studies of the 18-kDa translocator protein

**DOI:** 10.1007/s40336-015-0140-0

**Published:** 2015-09-10

**Authors:** Thalia F. van der Doef, Janine Doorduin, Bart N. M. van Berckel, Simon Cervenka

**Affiliations:** 1 Department of Psychiatry, University Medical Center Utrecht, Utrecht, The Netherlands; 2 Department of Radiology and Nuclear Medicine, VU University Medical Center, Amsterdam, The Netherlands; 3 Department of Nuclear Medicine and Molecular Imaging, University of Groningen, University Medical Center Groningen, Groningen, The Netherlands; 4 Department of Clinical Neuroscience, Centre for Psychiatry Research, Karolinska Institutet, Stockholm, Sweden

**Keywords:** Psychiatry, PET, Neuroinflammation, Microglia, TSPO

## Abstract

Accumulating evidence from different lines of research suggests an involvement of the immune system in the pathophysiology of several psychiatric disorders. During recent years, a series of positron emission tomography (PET) studies have been published using radioligands for the translocator protein (TSPO) to study microglia activation in schizophrenia, bipolar I disorder, major depression, autism spectrum disorder, and drug abuse. The results have been somewhat conflicting, which could be due to differences both in patient sample characteristics and in PET methods. In particular, further work is needed to address both methodological and biological sources of variability in TSPO levels, a process in which the use of animal models and small animal PET systems can be a valuable tool. Given this development, PET studies of immune activation have the potential to further increase our understanding of disease mechanisms in psychiatric disorders, which is a requisite in the search for new treatment approaches. Furthermore, molecular imaging could become an important clinical tool for identifying specific subgroups of patients or disease stages that would benefit from treatment targeting the immune system.

## The role of the immune system in psychiatric disorders

The concept that the immune system may have a role in the pathophysiology of psychiatric disorders has been discussed for several decades. Early observations in support of this hypothesis include reports of psychotic symptoms in autoimmune and infectious diseases engaging the central nervous system (CNS) [1, 2]. Furthermore, studies in both animals and humans show that immune activation can induce depressive-like symptoms, as part of what is commonly referred to as the “sickness behaviour” syndrome [3, 4].

With regard to studies in psychiatric populations, initial epidemiological observations of increased incidence rates of schizophrenia for cohorts born after influenza epidemics [5] have been followed up by a series of studies showing that infections during gestation or in early life lead to an increased risk of developing psychotic disorders [6]. In addition, autoimmune diseases and infections have been associated with mood disorders [7]. In support of a role for genetically determined alterations in the immune system in schizophrenia, a recent large-scale genome-wide association study found that associations were enriched for genes related to immune function [8]. Effects of genes related to immune function have also been found for depression, bipolar disorder, and autism [9, 10]. Furthermore, pharmacoepidemiological studies have suggested that anti-inflammatory drugs, such as non-steroidal anti-inflammatory drugs (NSAIDs), may lower the risk of psychiatric symptomatology [11].

Although the genetic and epidemiological data strongly suggest that immune function has a role in the development of psychiatric disease, an important question from a clinical perspective is if there are also ongoing brain immune disturbances in patients.

Post-mortem studies have shown increases in microglia in patients with schizophrenia [12, 13], autism [14], and cocaine abuse [15]. However, the post-mortem results may differ from the brain immune situation in vivo and in general there is a large delay between disease onset and post-mortem assessment. Studies on immune markers in blood, such as cytokines, have shown increased levels in both first-episode and chronic schizophrenia [16, 17] as well as in depression [18] and autism [19]. Similarly, gene expression of immune-related proteins has shown to be altered in schizophrenia and bipolar disorder [20]. However, findings have generally been inconsistent, with different markers showing effects in different studies, and there is often a large degree of overlap between patients and control subjects. Importantly, there is little direct passage of cytokines between brain and periphery [21, 22], and therefore, peripheral markers may not be representative of CNS processes. Studies in cerebrospinal fluid (CSF) are scarce, but alterations in immune markers have been shown for schizophrenia [23, 24], bipolar disorder [25, 26], and at least in a subgroup of patients with depression [27]. Although these studies are encouraging, a large degree of inconsistency still remains. Furthermore, CSF analyses cannot provide any anatomical information, and there is thus a need for more direct measures of brain immune activation in psychiatric patients.

## Measuring brain immune function in vivo: molecular imaging of TSPO

Molecular imaging is currently the only method to directly examine brain biomarkers at a molecular level in vivo. Of the available tools, positron emission tomography (PET) is the most accurate method, whereas single-photon emission computed tomography (SPECT) has been less prevalent due to the lower sensitivity as well as limited temporal and spatial resolution as compared to PET [28]. Thus far, all PET studies assessing the immune system in psychiatric populations have targeted the 18-kDa translocator protein (TSPO), and this method is thus the focus of the present review.

TSPO, formerly known as the peripheral benzodiazepine receptor, is located in the outer mitochondrial membrane of microglia and, to some extent, astrocytes. Although its exact function remains unclear, TSPO has been described to be involved in many physiological processes, including cholesterol transport and steroid synthesis [29]. Importantly, microglia are considered to be the key cell type involved in CNS immune processes, as they can be activated by cytokines and additionally contribute further to cytokine release [30]. Activation of resident microglia and infiltration of macrophages contribute to neuronal injury and synaptic damage [29], although neurotrophic and protective functions have also been observed [31, 32]. TSPO is a widely used marker for immune activation in the CNS, since a change in phenotype and functional state of microglia is associated with increased TSPO expression [30].

The PET radioligand (*R*)-[^11^C]PK11195 has been the most commonly used PET tracer to assess TSPO in the CNS. Several studies describe prolonged increased binding in patients with stroke and traumatic brain injury which can be seen as proof-of-concept studies [33, 34]. Since(*R*)-[^11^C]PK11195 shows a high level of non-specific binding, during the last decade significant efforts have been made to develop new TSPO radioligands. Those that have hitherto been applied to psychiatric conditions are [^11^C]DAA1106, [^18^F]FEPPA, and [^11^C]PBR28 [35–40]. These second-generation ligands are considered to have improved signal-to-noise ratio compared to [^11^C]PK11195, based on a 7- to 60-fold higher affinity for TSPO and animal blocking studies showing up to 80-fold higher specific binding than [^11^C]PK11195 [36, 38, 40, 41]. A practical drawback with all these novel compounds is their sensitivity to a polymorphism in the gene encoding TSPO, which leads to three distinct binding classes: high-affinity binders (HABs; 49 % of the Western population), mixed-affinity binders (MABs; 42 %), and low-affinity binders (LABs; 9 %) [13, 42]. As a consequence, up to ten percentage of the population cannot be part of clinical studies due to LABs. In the remaining subjects, the effects of TSPO polymorphism require genotyping the subjects and controlling for genotype in the study design and analysis. Furthermore, second-generation TSPO radioligands have thus far not been validated in preclinical models to the same extent.

## Psychiatric disorders and TSPO PET imaging: clinical studies

We identified all PET studies that examined TSPO binding in psychiatric disorders (Table 1). Thus far, schizophrenia is the most studied condition with four articles, whereas only one to two studies have been published for mood disorders, autism spectrum disorder, and drug abuse.

**Table 1:** Overview of clinical TSPO PET studies in psychiatric disorders

References	Disorder	Tracer	Controls/patients (number)	PET outcome measure	Main findings	Regions studied
Kenk et al. [46]	Schizophrenia	[^18^F]FEPPA	27/16	2TCM *V*_T_	No group differences	HIPP, TC, mPFC, DLPFC, striatum, CC, cingulum, PLIC, SLF
Takano et al. [45]	Schizophrenia	[^11^C]DAA1106	14/14	2TCM BP_ND_*	No group differences; correlations with symptom severity and disease duration	MTC, LTC, PC, OC, CER, striatum, ACC, PCC
Doorduin et al. [44]	Schizophrenia	[^11^C]PK11195	8/7	2TCM BP_ND_	Increase in hippocampus	HIPP, TC, FC, PC, OC, CER, basal ganglia, TH, midbrain, pons
van Berckel et al. [43]	Schizophrenia	[^11^C]PK11195	10/10	2TCM BP_P_	Increase in total grey matter	Total grey matter
Setiawan et al. [53]	Major depression	[^18^F]FEPPA	20/20	2TCM *V*_T_	Increases in all regions; correlations with symptom severity	HIPP, TC, MPFC, DLPFC, VLPFC, FP, PC, OC, dPU, dCA, VST, ACC, TH, insula
Hannestad et al. [50]	Major depression	[^11^C]PBR28	10/10	1TCM, 2TCM, MA1 *V*_T_	No group differences	TC, FC, PC, OC, CER, TH, CA, PU, white matter
Haarman et al. [55]	Bipolar I disorder	[^11^C]PK11195	11/14	2TCM BP_ND_	Increase in right hippocampus	HIPP, TC, FC, DLPFC, PC, OC, CB, ACC, PCC, basal ganglia
Suzuki et al. [59]	Autism spectrum disorder	[^11^C]PK11195	20/20	SRTM BP_ND_ Ref: cerebellum**	Increase in all regions	STC, fusiform cortex, MFC, OFC, PC, CER, ACC, CC, brainstem
Narendran et al. [61]	Cocaine abuse	[^11^C]PBR28	17/15	2TCM *V*_T_	No group differences	MTL, DLPFC, MPFC, OFC, CER, ACC, ST, midbrain
Sekine et al. [60]	Methamphetamine abuse	[^11^C]PK11195	12/12	SRTM BP_ND_ Ref: frontal, parietal, occipital cortex**	Increase in all regions	OC, insular cortex, TH, ST, midbrain

*1TCM* one-tissue compartment model, *2TCM* two-tissue compartment model, *MA1* multilinear analysis, *SRTM* simplified reference tissue model, *BP*_*ND*_ binding potential, *V*_*T*_ total distribution volume, *d* dorsal/dorso, *l* lateral, *m* medial, *o* orbito, *p* pre, *s* superior, *v* ventral/ventro, *ACC* anterior cingulate cortex, *CA* caudate, *CER* cerebellum, *CC* corpus callosum, *FC* frontal cortex, *FP* frontal pole, *HIPP* hippocampus, *OC* occipital cortex, *PC* parietal cortex, *PCC* posterior cingulate cortex, *PLIC* posterior limb of the internal capsule, *PU* putamen, *SLF* superior longitudinal fasciculus, *ST* striatum, *TH* thalamus, *TC* temporal cortex

* TSPO genotype was not accounted for

** Ref = reference tissue

### Schizophrenia

The first study on TSPO in schizophrenia used the radioligand (*R*)-[^11^C]PK11195 in ten patients and ten age-matched healthy control subjects [43]. All patients were examined within 5 years of disease onset, with an average disease duration of 3.1 ± 1.7 years. (*R*)-[^11^C]PK11195 binding potential (BP_P_, as calculated with K1*k3/k2*k4) values were calculated using a two-tissue compartment model (2TCM) with metabolite-corrected plasma as input function. Patients had mild symptoms at the time of PET scanning as measured with the Positive and Negative Syndrome Scale (PANSS), with average scores of 12 ± 3 and 14 ± 4 for symptoms, respectively, and they were all on atypical antipsychotics. The authors observed an increase in (*R*)-[^11^C]PK11195 binding potential in patients in total grey matter, which was the only region analysed (Table 1). No significant correlation between symptom severity and total grey matter BP_P_ was found.

Doorduin et al. [44] used (*R*)-[^11^C]PK11195 to examine seven patients with schizophrenia with active psychosis, defined by a score of 5 or more on 1 PANSS positive symptom item or a score of 4 on 2 items, in comparison with eight healthy volunteers. The disease duration ranged from 1 to 16 years (average 5.3 ± 5.6 years), with 1–4 experienced psychotic episodes. Patients had moderate symptoms at the time of PET scanning, with average PANSS scores of 20 ± 3, 17 ± 5, and 37 ± 7 for positive, negative, and general subscales, respectively. All patients were using antipsychotics, and the use of benzodiazepines was allowed for 1–2 weeks prior to PET examinations. Substance use and anti-inflammatory drugs were reported as exclusion criteria. (*R*)-[^11^C]PK11195 binding potential (BP_ND_ defined as k3/k4) was quantified using 2TCM. In contrast to the study by van Berckel et al. [43], the (*R*)-[^11^C]PK11195 binding potential of whole-brain grey matter was not found to be increased in patients compared to control subjects. Multiple ROIs were examined (Table 1), and a significant increased BP_ND_ was found in the hippocampus of patients. To reduce the variation in the small sample size, the data were normalised to the whole-brain grey matter for statistical analysis which means that the results are not directly comparable to the other TSPO studies in schizophrenia.

Takano et al. [45] studied fourteen patients with chronic schizophrenia and fourteen age- and sex-matched controls with the second-generation tracer [^11^C]DAA1106. Patients had a long disease duration (18.8 ± 12.2 years). All patients were on antipsychotics, and benzodiazepines were allowed for more than 1 month before the start of the study. Substance and alcohol abuse were exclusion criteria. All cortical grey matter regions were assessed, as well as the striatum, and BP_ND_ as quantified using 2TCM was the main outcome measure. Patients had moderate symptoms at the time of PET scanning as scored on PANSS (total score 78.6 ± 20.7). Although no significant differences in TSPO levels between patients and controls were reported, correlations were found between TSPO binding and disease duration as well as positive symptoms. However, TSPO genotype was not determined, and since in vitro studies show fourfold differences in [^11^C]DAA1106 affinity between MAB and HAB subjects [42], this significantly limits the interpretation of the results.

Using [^18^F]FEPPA, Kenk et al. [46] examined grey matter frontal and temporal ROIs, striatum, and white matter ROIs in a sample of 16 patients (10 HABs and 6 MABs) and 27 controls (19 HABs and 8 MABs; Table 1). The disease duration was 14.8 ± 8.8 years, and patients had moderate symptoms at the time of PET scanning (PANSS total 70.2 ± 9.7). Patients were on treatment with either atypical or typical antipsychotics, and a minor proportion were also using antidepressants or anti-Parkinsonian drugs. Benzodiazepine use was not allowed except for clonazepam. Total distribution volumes (*V*_T_) values were calculated using 2TCM with an arterial plasma input function. Since [^18^F]FEPPA binding is also sensitive to TSPO genotype, this factor was included in the statistical analysis. No significant differences in *V*_T_ values were found between patients and controls in either white or grey matter regions.

Clinical factors that might influence the study results are patient characteristics such as disease duration, symptom severity, and medication use. Both the two (*R*)-[^11^C]PK11195 studies that found an effect had patients with a shorter disease duration [43, 44], whereas no difference was reported in patients with a longer disease duration [46]. This could indicate that microglia activation has a more prominent role in early disease phases. However, in order to answer this question longitudinal studies are necessary, ideally including also measurements in first-episode patients and high-risk individuals. In addition, patients had mild to moderate symptoms at the time of PET scanning, and thus it is of interest to examine if more severe pathology shows a greater increase in TSPO binding. Importantly, benzodiazepine use was an exclusion criterion in all studies (except for clonazepam in one study [46]) since most benzodiazepines have affinity for the TSPO and can compete for binding with TSPO with the PET tracer [47]. However, a recent study found that this was mainly the case for the benzodiazepine diazepam in higher doses [48]. Further studies should be performed to confirm this observation, as alleviating this restriction could potentially improve recruitment of more severely ill patients.

One important limitation in all studies published thus far is that all patients were on treatment with antipsychotic drugs. Most antipsychotics, besides clozapine, tend to decrease TSPO expression [49]. Although evidence from in vivo human research is lacking, this suggests that there might be an underestimation of the signal. For instance, this could explain the negative findings in Kenk et al. [46]. Examination of drug-naive patients will be critical to address this issue.

### Mood disorders

To date, two studies assessed TSPO in patients with major depressive disorder and one study examined bipolar I patients (Table 1).

In the study by Hannestad et al. [50], [^11^C]PBR28 was used to examine ten patients with major depression (MD) and ten healthy controls. The sample included seven HABs in both the patient group and control group, respectively. Patients were allowed to take antidepressant medications if the dose had been stable for at least 4 weeks (although it was not reported how many patients were administered antidepressants at the time of PET). Laboratory signs of peripheral immune activation, as defined by elevated high-sensitive C-reactive protein (CRP), were an exclusion criterion. Symptom severity at the time of examination was mild to moderate, with 19.7 ± 6.7 scores on the Montgomery–Åsberg Depression Rating Scale (MADRS, range 0–60; 0–6 normal, 7–19 mild, 20–34 moderate, >34 severe depression [51]). This represented a significant reduction from 25.6 ± 7.5 at screening. Groups were matched for TSPO genotype. [^11^C]PBR28 *V*_T_ values were calculated using 1TCM, 2TCM, and the multilinear analysis MA1 [52], using the metabolite-corrected arterial plasma curve as input function. In addition, *V*_T_ corrected for plasma free fraction (*V*_T_/f_P_) was determined. No significant differences in binding were found in a selection of cortical ROIs, white matter, and basal ganglia. Using *V*_T_/f_P_ instead of *V*_T_ did not change these results.

Setiawan et al. [53] examined twenty MD patients (15 HABs, 5 MABs) and twenty healthy controls (14 HABs and 6 MABs) using [^18^F]FEPPA. All patients were medication-free for at least 6 weeks prior to the PET examination. Depressive symptoms were moderate to severe, with average scores of 20 ± 3.8 on the 17-item Hamilton Depression Rating Scale (HDRS, range 0–52; 0–7 normal, 8–13 mild, 14–18 moderate, 19–22 severe, ≥23 very severe depression [54]). [^18^F]FEPPA *V*_T_ calculated using 2TCM was the main outcome measure, and ROIs selected were prefrontal cortex, anterior cingulate cortex (ACC), and insula. Genotype was included as a factor in the statistical analysis. A global effect of diagnosis on [^18^F]FEPPA *V*_T_ was shown, with higher values in patients in all regions examined. Furthermore, HDRS scores were positively correlated with TSPO *V*_T_ in the ACC, after correcting for genotype. Serum markers for neuroinflammation were measured, but there were no correlations with brain TSPO levels.

Haarman et al. [55] studied fourteen bipolar I patients compared to eleven healthy volunteers. All patients except one were euthymic, and the disease duration ranged from 2 to 37 years (average 25.6 ± 12.0 years). All patients had experienced multiple depressive and hypomanic or manic episodes. All except one patient were on mood-stabilising medication. (*R*)-[^11^C]PK11195 BP_ND_ was quantified using 2TCM with plasma input function. Multiple brain regions were analysed, although hippocampus was selected as the main ROI, based on human and preclinical evidence suggesting that this region could be a particular focus of microglia activation. For the statistical analysis, binding in whole-brain grey matter was used as a covariate to reduce the between-subject variation. Whereas BP_ND_ in the whole-brain was not different between patients and healthy volunteers, higher values were observed in the right hippocampus of bipolar I patients.

The differences in clinical factors between the mood disorder studies make it difficult to compare the PET outcome. In the two studies on depression, the study of Setiawan et al. [53] had a significantly larger sample size, and patients had more severe symptoms than in the study of Hannestad et al. [50], which might explain the difference in results. A further difference is the exclusion of signs of mild peripheral immune activation in the latter study. Since CRP has shown to be elevated in patients in depression [56], suggesting that peripheral immune activation is related to the disease mechanism, this could have led to a selection bias towards patients with relatively low levels of immune activation.

Furthermore, in two of the studies the patients were on treatment with mood-stabilising medication [50, 55], whereas in the study by Setiawan [53], patients were medication-free for 6 weeks. Most mood-stabilising medications tend to decrease TSPO expression [57, 58], which suggests an underestimation of the signal in the patients who were assigned to treatment, although this has to be confirmed in future studies with medication-naïve patients.

### Developmental disorders

To date, only one PET study has assessed TSPO binding in patients with developmental disorders. In the study by Suzuki et al. [59], (*R*)-[^11^C]PK11195 was used to examine twenty individuals with autism spectrum disorder and twenty age- and intelligence quotient (IQ)-matched controls. Patients were young adults in an age range from 18.6 to 31.9 (average 23.3 ± 4.0) years, had IQ scores above 80 (average 95.9 ± 16.7), and did not receive any medication. Arterial blood was not sampled; instead, (*R*)-[^11^C]PK11195 BP_ND_ was quantified using a reference tissue approach. A normalised input curve based on the averaged TACs from the ROIs placed over the cerebellar cortices in the control group was used for both the control and the patient data analysis. In the ROI-based analysis, an overall statistical effect was shown, and increased (*R*)-[^11^C]PK11195 binding potential was reported in all examined regions, including (subparts of) the cerebellum, ACC, corpus callosum, and frontal, temporal, and parietal cortex (Table 1). These results were confirmed in a voxel-based analysis. There were no correlations between TSPO BP_ND_ and clinical symptoms levels. As discussed below, the reference tissue approach used in the study limits the conclusions that can be drawn.

### Substance use disorders and the potential confound of drug use

Two PET studies examined TSPO in substance use disorders (Table 1). Sekine et al. [60] used (*R*)-[^11^C]PK11195 to examine twelve methamphetamine abusers in comparison with twelve healthy volunteers. The disease duration of the patients ranged from 1 to 12 years (average 6.8 ± 3.9 years); however, patients were abstinent on average almost 2 years prior to PET examinations. A modified version of the Drug Effect Rating Scale was used to assess the scale for methamphetamine craving. Scores were in a range from 1 (no craving) to 10 (most intense craving) with an average score of 4.9 ± 3.4. PET data were analysed with a reference tissue approach, whereby averaged TACs from the frontal, parietal, and occipital cortices from the control group were used for both the control and the patient data analysis. Significantly higher BP_ND_ values were found in methamphetamine abusers in all ROIs studied, with differences ranging from 3- to 15-fold. A significant negative correlation was found between (*R*)-[^11^C]PK11195 BP_ND_ and the duration of methamphetamine abstinence in midbrain, thalamus, and striatum, suggesting that microglial function can be normalised by protracted abstinence.

In the study by Narendran et al. [61], 15 chronic cocaine abusers (8 HABs, 5 MABs, 2 LABs) and seventeen healthy controls (12 HABs, 4 MABs, 1 LAB) were examined using [^11^C]PBR28. Patients were on average 39.9 ± 9.0 years old and had been smoking crack cocaine for an average of 17 ± 7 years. Patients had a minimum of 2 weeks outpatient abstinence as monitored with urine toxicology and did not receive medication. *V*_T_ values were calculated in a standard manner, using 2TCM with an arterial plasma input function, and data were corrected for TSPO genotype. No differences in [^11^C]PBR28 *V*_T_ were observed between patients and control subjects, as assessed in both cortical and subcortical brain regions.

Thus, these two studies differ markedly in their findings. However, apart from the fact that different drugs of abuse were studied, a direct comparison is precluded since there are major differences in both the clinical status of patients, such as time of abstinence, and methodology—in particular, the use of a reference region in the study by Sekine et al. (see below for discussion).

For most psychiatric patients groups, there is an increased prevalence of substance use disorders, such as alcohol, cannabis, and nicotine use [62]. For instance, cannabis is considered to be the most commonly used illegal drug of abuse among patients with bipolar disorder and schizophrenia [63, 64]. Although substance use disorder was an exclusion criterion in all studies, it cannot be excluded that patients had a higher recreational use of drugs of abuse. In most studies, actual drug use was assessed with urine analysis [43, 46, 50, 53, 60, 61]. However, only four of the studies reported on or controlled for smoking status, of which one study included only non-smokers [53], one study reported only three smokers in the patient group [50], one study allowed smoking and matched patients and controls for nicotine smoking status [61], and one study did not include subjects that fulfilled nicotine-related DSM-IV criteria [60]. Importantly, in vitro studies have shown that cannabinoids and nicotine have a suppressant effect on the immune system [65, 66]. Although these observations remain to be confirmed in vivo, the use of these common drugs of abuse could thus theoretically diminish differences in TSPO levels between patients and control subjects, which might in some cases contribute to negative findings. In contrast, if an effect of psychostimulants in TSPO levels even after long abstinence intervals can be confirmed, group differences in the use of this drug could confound the results in the other direction.

## Preclinical studies of TSPO and psychiatric disorders

### Animal models and microglia activation

As psychiatric disorders are complex human disorders of which the aetiology and the exact pathophysiology are unknown, the three criteria commonly used for the validation of an animal model, i.e. face, construct, and predictive validity [67], cannot be fulfilled by a single animal model. Animal models of psychiatric disorders do therefore only manifest certain aspects that can be translated to the human disease, i.e. endophenotypes, such as hyperlocomotion, anxiety, or deficits in prepulse inhibition. With regard to the role of the immune systems in psychiatric disorders, many animal models have linked psychotic- and depressive-like behaviour to inflammatory processes. For instance, modelling effects of maternal immune activation on the risk of developing schizophrenia, viral infection of pregnant dams resulted in psychotic-like symptoms in the offspring, and also microglia were found to be activated [68, 69]. The bacterial endotoxin lipopolysaccharide (LPS) is often used to induce depressive-like behaviour in rodents, leading to immediate sickness behaviour followed by behavioural deficits that can be linked to depression [70, 71]. Importantly, LPS has been shown to induce an activation of microglia, lasting several days, as detected using immunohistochemistry [72]. Conversely, animal models for stress-induced depression have shown to induce both depressive behaviour and microglia activation [73].

### Preclinical TSPO PET imaging

**Fig. 1:**
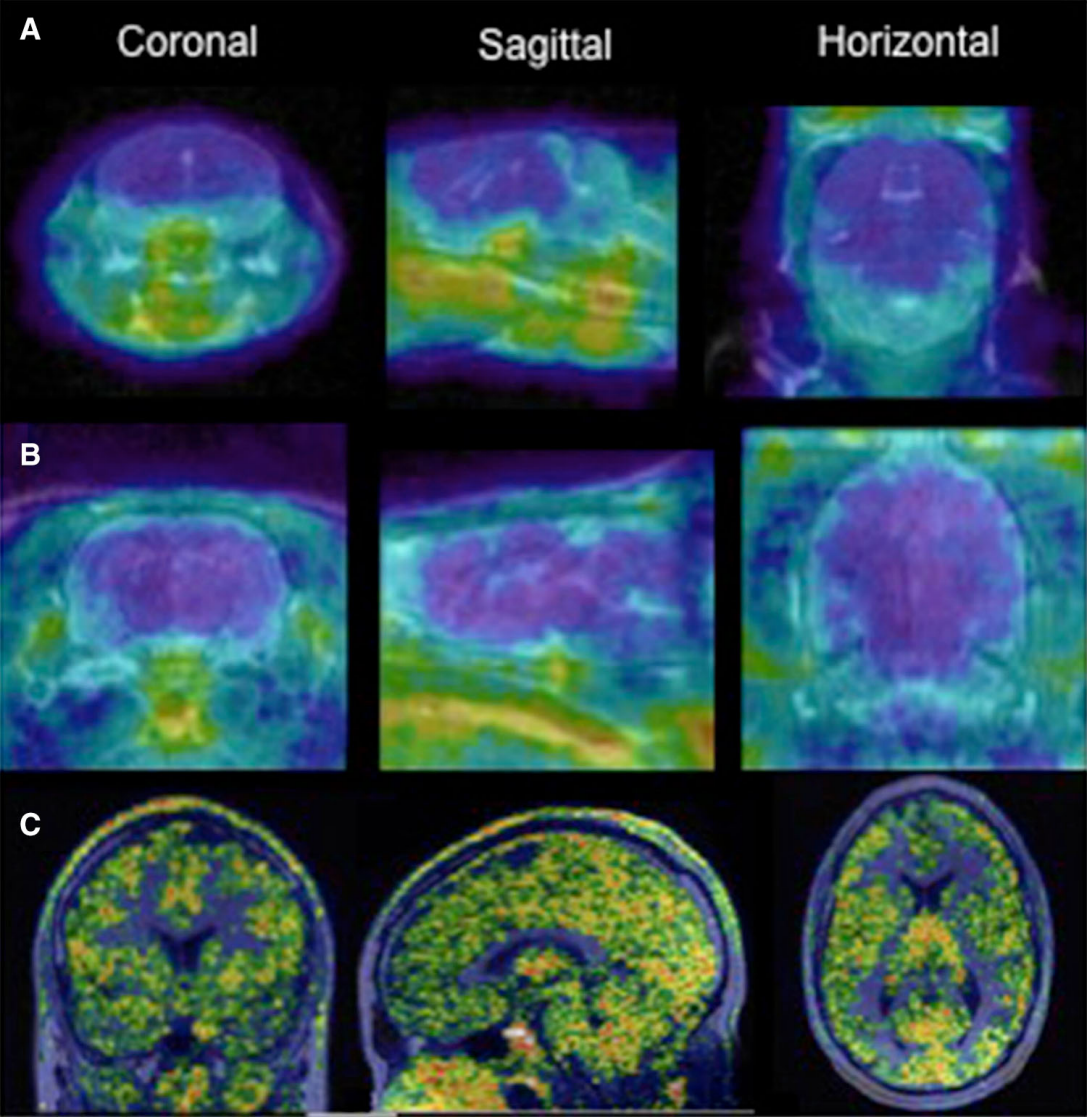
Cross-species TSPO imaging using [^11^C]PBR28: SUV images of **a** mouse **b** rat, and **c** a healthy human subject. All PET images are overlaid over MR images. Courtesy of the PET centre, Karolinska Institutet

With the advent of small animal PET systems, it has become feasible to estimate TSPO levels in rats and mice, thus enabling a translational approach (Fig. 1). Thus far, only a few studies have assessed TSPO in animal models of psychiatric disorders. Herpes virus-induced psychotic-like behaviour in rats was found to be accompanied by microglia activation, as shown with (*R*)-[^11^C]PK11195 PET [74]. (*R*)-[^11^C]PK11195 uptake was found to increase with the increased severity of psychotic-like behaviour, and psychotic-like behaviour and microglia activation could be reduced by antipsychotic treatment. Dobos et al. [75] used (*R*)-[^11^C]PK11195 PET to show microglia activation and depressive-like behaviour following intracerebral LPS injection in mice. Recently, LPS-induced systemic inflammation in baboons was shown to induce an activation of microglia, as shown with [^11^C]PBR28 PET [76]. A robust increase in TSPO binding was observed at 1 and 4 h after intravenous injection of LPS. Depressive-like behaviour was not assessed in this study.

## Methodological considerations

Taken together, the data thus far suggest a modest elevation of TSPO in psychiatric disorders. In general, the increase was found throughout the entire brain, although some studies reported a more focal elevation. However, almost half of the studies did not find a difference between patients and control subjects. Importantly, the results of the TSPO PET studies are in many instances difficult to compare, not only due to differences in patient characteristics as discussed above, but also since the methods differ in many aspects. In the following, we discuss some key methodological points of relevance for interpreting the findings.

The gold standard for TSPO PET quantification is to apply a 2TCM, using a metabolite-corrected plasma input function to estimate the contribution of free and non-specific binding to the brain signal. The outcome parameter of choice using this approach is *V*_T_, since the direct estimation of BP, for instance, by calculating k3/k4 is less reliable. Therefore, to facilitate comparisons between the studies, it would have been useful if the studies with BP as outcome parameter had additionally reported *V*_T_ values.

Plasma input models are considered to have a high variability, which may be due to methodological issues in measuring the metabolite-corrected plasma input curve. In order to reduce variability of plasma input models, several approaches have been proposed.

In two of the studies reviewed above, normalisation of regional values to whole-brain binding was performed [44, 55]. Although this reduces the variance, the drawback with this approach is that it may underestimate true specific binding, especially in case of a global increase. Furthermore, the whole cortex cannot be analysed using this approach.

Reference tissue approaches, such as simplified reference tissue method (SRTM) [77] and Logan DVR [78], are in general preferred above plasma input models if a reference region can be identified, both to reduce noise as well as from a practical perspective since arterial sampling can then be eliminated. This may be particularly important for psychiatric patient groups. One method is to use ratios of standardised uptake values, which was recently reported to be more sensitive than *V*_T_ to detect TSPO elevations in Alzheimer’s disease for [^11^C]PBR28, with cerebellum as reference region [79]. However, the use of this method assumes a very focal TSPO elevation, which might not be the case for most psychiatric disorders, and is sensitive to bias induced by difference in blood flow between regions and between groups.

In general, all reference tissue approaches are problematic in TSPO PET studies as there is no anatomical region in the brain devoid of TSPO. Whereas this protein was initially not thought to be present in the non-diseased brain, widespread (*R*)-[^11^C]PK11195 BP_ND_ increase was found in healthy ageing [80]. In addition, specific binding to TSPO in healthy individuals was recently confirmed in a TSPO blocking study [81]. In the studies on autism and methamphetamine use by Suzuki et al. [59] and Sekine et al. [60], normalised average time activity curves (TACs) from the control group were used for both patient and control data, which is not according to convention for reference tissue approaches and creates further problems. In this case, group differences in radioligand delivery and non-displaceable binding are not taken into account, which might generate artificially high BP_ND_ values in patients, and the results therefore have to be interpreted with caution.

An alternative approach to identifying a reference “region” is to identify voxels with low specific binding based on the shape of the TAC using supervised cluster analysis. Several methods have been published for this purpose, and they show reliable results in normal ageing and after traumatic brain injury, both conditions leading to robust microglia activation [80, 82]. However, the validity of voxels devoid of TSPO can be questioned as for anatomical reference regions, and this method might underestimate the signal in comparison with other approaches [83]. Another method to eliminate the need for arterial blood sampling is to use an image derived input function, by extracting time-activity curves from PET voxels corresponding to blood vessels. This approach has been validated for tracers such as [11C] flumazenil and [11C] AZ10419369 [84], and preliminary data has shown feasibility for this method to quantify [11C]PBR28 using HRRT [85]. So far, neither of these approaches have been used to study psychiatric populations.

## General comments and future directions

It is well established that immune activation generally has a negative effect on psychological well-being and may contribute to both psychotic and depressive symptoms. A role for immune-related processes as part of specific, causative disease mechanisms in psychiatric disorders is at present more uncertain. For schizophrenia and autism, both genetic and epidemiological associations to immune function could reflect a role for immunological factors affecting neurodevelopment, which, for instance, is in line with recent observations suggesting that microglia are involved in synaptic pruning as well as providing trophic support for neuronal growth [32]. An alternative interpretation of these early effects is the “sensitisation” theory, postulating that early immune challenges to a genetically vulnerable system lead to an increased inflammatory response later in life [86]. Even so, the changes in psychiatric disorders are likely to be more subtle than for classic CNS autoimmune disorders such as multiple sclerosis (MS) and systemic lupus erythematosus (SLE), as there are no radiological signs and the changes in CSF and blood are less distinct. Since the immune system can in certain instances even be regarded as neuroprotective [31], immune dysregulation in psychiatric disorders may even be conceived as an imbalance rather than a “one-dimensional” immune response.

Given these proposed more subtle immune changes in psychiatric disorders, a major challenge for clinical TSPO PET studies is the large degree of variability even in healthy individuals. This is true irrespective of the radioligand and methods of quantification used. In the studies reviewed above, where variability was reported, the overall % covariance (COV) was around 35 % for second-generation radioligands across genotypes, and 25 % for (*R*)-[^11^C]PK11195. With regard to interindividual variability, this has shown to be moderate to high for both (*R*)-[^11^C]PK11195 [87] and [^11^C]PBR28 [88, 89], whereas reproducibility has not been assessed for any other TSPO radioligands. Although part of this variability may be due to methodological noise as discussed above, there may also be biological explanations. For instance, in Collste et al. [89] diurnal effects were shown on GM *V*_T_ values, indicating that part of the variability could be due to natural fluctuations in the immune system in health. The variability in TSPO levels has important implications in terms of the sample samples needed, both for group comparisons and for longitudinal studies. For instance, in the study on schizophrenia by Kenk et al. [46] based on mean and variability of VT values, it was calculated that 21 subjects per group was required to detect a 20 % difference (α = 0.05 and power = 0.8), which can be compared to the 16 % increase in [^3^H]PBR28 shown in a post-mortem study [13]. If similar effects are expected in other disorders, most studies performed thus far may have been underpowered. Sample size may be particularly critical for studies using second-generation radioligands, since accounting for TSPO genotype in the analysis reduces statistical degrees of freedom. To enhance the sensitivity of TSPO PET, an important direction of research will be not only to reduce methodological noise by finding reliable alternatives to arterial sampling, but also to identify the physiological factors that may influence TSPO levels in brain.

One important limitation for PET TSPO studies in general is the lack of specificity of this marker. For instance, TSPO is present also in astrocytes, which could contribute to the signal [90, 91], and although both cell types contribute to immune response, the specific type of cell may have implications in terms of treatment. Furthermore, for the microglia population, TSPO cannot distinguish between pro-inflammatory (M1) and protective (M2) subpopulations. Thus, it will also be crucial to developing tracers for other, more specific microglia markers that are also sensitive to the functional phenotype. Importantly, PET data then need to be combined with other measures of immune function in order to fully reflect the immunological status for each disease condition. Although TSPO levels were compared to serum markers of inflammation in some studies reviewed above, ideally this comparison should be done using CFS samples which more closely reflect the state of the CNS immune system.

A crucial aim for research on the immune system in psychiatry is to inform new treatment strategies. In schizophrenia, depression, and autism, randomised controlled studies have shown that the addition of cyclooxygenase (COX)-2 inhibitors to antipsychotics and antidepressants treatment may lead to amelioration of symptoms [92–94], although negative results have also been reported [95]. In psychosis, both the COX inhibitor aspirin and the broad-spectrum tetracycline antibiotic minocycline, which directly attenuates microglia activation, have shown to reduce symptoms [96, 97]. However, the immune hypothesis of psychiatric disorders would implicate a need for drugs specifically directed towards causative mechanisms. Molecular imaging is an important tool towards this goal, as it allows for non-invasive studies in both patients and animal models. Thus, immune markers can be followed over time, allowing for monitoring of disease progress and treatment effect. For instance, [^11^C]PBR28 PET was recently used to detect effects of immune-targeted treatment in Parkinson’s disease [98]. Furthermore, due to the translational nature of PET, clinical studies can be supported by preclinical experiments, also allowing for validation with post-mortem techniques such as immunohistochemistry. For many psychiatric disorders where large clinical trials have suffered from negative findings, these approaches could be critical towards revitalising drug development programmes.

With increasing understanding of the biological processes as well as methodological refinement, PET studies of the immune system promise to be an important tool also for clinical use. An important task will be to aid diagnosis and potentially identify specific subgroups of psychiatric patients in need of immune-targeting treatment. For instance, a proportion of patients with schizophrenia show auto-antibodies for neural receptors, suggesting that these patients may constitute a subgroup in specific need of treatment targeted against the immune system [99]. In depression, specific subgroups of patients were shown to respond to anti-TNF treatment [100]. Importantly, such PET markers may prove to be specific for particular pathophysiological mechanisms and symptom dimensions, rather than reflecting current disease classifications according to DSM.
